# Predictors of early colorectal cancer metastasis to lymph nodes: providing rationale for therapy decisions

**DOI:** 10.3389/fonc.2024.1371599

**Published:** 2024-07-05

**Authors:** Xu Song, Jun Li, Jiang Zhu, Yun-Fei Kong, Yu-Hang Zhou, Zi-Kun Wang, Jin Zhang

**Affiliations:** Department of General Surgery, Affiliated Hospital of Jiangsu University, Zhenjiang, Jiangsu, China

**Keywords:** T1 colorectal cancer, lymph node metastasis, predictive factor, additional surgery, artificial intelligence

## Abstract

With the improvement of national health awareness and the popularization of a series of screening methods, the number of patients with early colorectal cancer is gradually increasing, and accurate prediction of lymph node metastasis of T1 colorectal cancer is the key to determining the optimal therapeutic solutions. Whether patients with T1 colorectal cancer undergoing endoscopic resection require additional surgery and regional lymph node dissection is inconclusive in current guidelines. However, we can be sure that in early colorectal cancer without lymph node metastasis, endoscopic resection alone does not affect the prognosis, and it greatly improves the quality of life and reduces the incidence of surgical complications while preserving organ integrity. Therefore, it is vital to discriminate patients without lymph node metastasis in T1 colorectal cancer, and this requires accurate predictors. This paper briefly explains the significance and shortcomings of traditional pathological factors, then extends and states the new pathological factors, clinical test factors, molecular biomarkers, and the risk assessment models of lymph node metastasis based on artificial intelligence.

## 1 Introduction

Colorectal cancer (CRC) is the third most common cancer worldwide with an incidence rate of about 10.0% and is the second leading cause of cancer-related death (1). At present, a growing number of patients with CRC can be found at an early stage with the improvement of national health awareness and the popularization of a series of screening methods (2). Even with the mature application of endoscopic technology, surgical resection is still the first treatment that comes to our mind when dealing with CRC. However, surgical operations face many problems, such as a high rate of postoperative complications or decreased quality of life (3). Under such circumstances, the steady development of endoscopic technology provides new ideas for the treatment of early CRC, but this local excision is oncologically safe only in the absence of lymph node metastasis (LNM) (4). Previous studies have found that the overall survival rate and disease-free survival rate of early CRC with LNM are significantly lower than those without LNM (5), and the disease of patients with LNM is often able to progress to a later stage.

The early CRC can be divided into intramucosal (pTis) and submucosal (pT1) ones, which has been defined as a carcinoma that penetrates the muscularis mucosae layer and infiltrates into the submucosa, regardless of lymph node status (6). It is reported that intramucosal CRC has almost no risk of LNM, which is a clear indication of endoscopic resection (7). On the other hand, in patients with submucosal invasive CRC, the risk of LNM is 7.0%-16.9% (8–10). At this time, local resection alone cannot effectively prolong the life of patients, and such patients often require additional bowel resection and lymph node dissection after endoscopic resection to cure (11). Of course, the guidelines of each country have their criteria for additional secondary surgery. But even if these guidelines are followed, the LNM rate of patients undergoing secondary surgery only increases to 7.3%-15.5% (12, 13). That is to say, more than 80% of patients with early CRC without LNM have undergone surgery, and this has not brought better clinical benefits (14, 15). To screen out patients without LNM in T1 CRC cases better, more accurate methods are needed to predict the risk of LNM in early-stage CRC patients. In this article, we provide an overview and analysis of the risk factors for the presence of LNM in pT1 CRC that have been newly proposed in recent studies.

## 2 Pathological factors included in the guidelines and their shortcomings

The high-risk factors related to LNM of early CRC mentioned in the current national guidelines mainly include the depth of submucosal infiltration, lymphovascular invasion, histological grade, and tumor budding (**Table 1**). These traditional pathological factors’ definitions, significance, and shortcomings will be discussed below.

**Table 1:** Pathological factors in the guidelines.

	JSCCR (7)	NCCN (16)	ESMO (17, 18)	ESGE (19)
Depth of submucosal invasion	√	√	√	√
Lymphovascular invasion	√	√	√	√
Histological grade	√	√	√	√
Budding grade	√		√	√

JSCCR, Japanese Society for Cancer of the Colon and Rectum guidelines; NCCN, National Comprehensive Cancer Network; ESMO, European Society for Medical Oncology; ESSGE, European Society of Gastrointestinal Endoscopy. The √ indicates the pathological factors included in the guidelines.

### 2.1 Depth of submucosal infiltration

**Figure 1:**
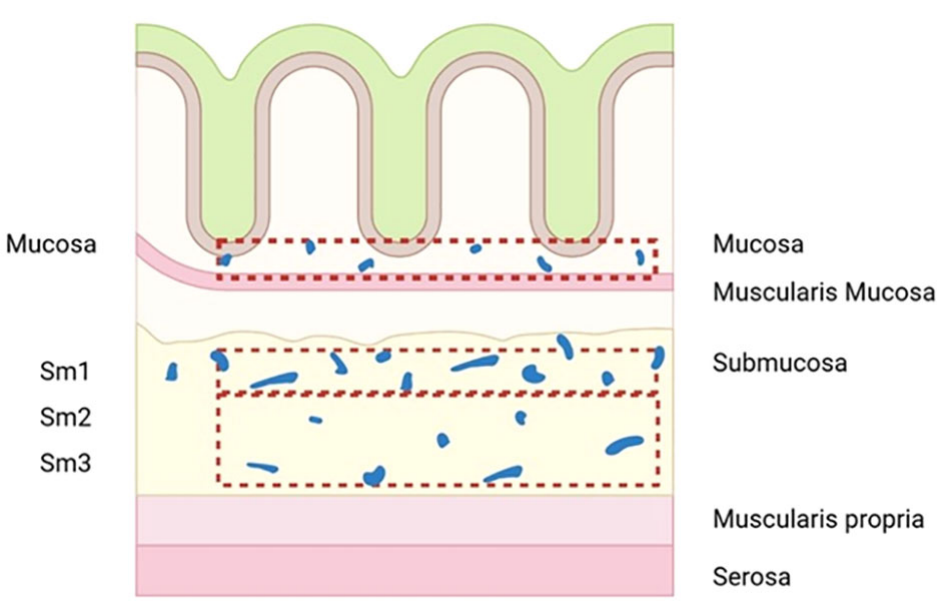
Diagram to illustrate the number of lymphatic vessels in the superficial third of the submucosa (sm1) is significantly more than in deeper layers (sm2, sm3).

The correlation between the depth of submucosal infiltration (DSI) and LNM in early CRC has been verified in many studies (7, 17, 20, 21). KuDo divided submucosa into three layers: superficial layer, middle layer, and deep layer (sm1, sm2, and sm3), and a further study by Nascimbeni et al. showed that the risk of LNM increased with deepening SM grade (22). However, this index may be affected by factors such as specimen quality and histological technical preparation, eventually leading to large individual differences. In addition to this, an increasing number of authors currently question its validity (23–27). They found that the number of lymphatic vessels in the superficial third of the submucosa (sm1), which had a relatively low risk of LNM, was significantly more than that in the deeper layers (sm2, sm3) (**Figure 1**) (28). This indicates that tumors invading only the superficial third of the submucosa but with a large area of invasive margin may be more likely to enter the lymphatic system than tumors that are narrow but invade sm3 deeply (29), which was also confirmed in a recent retrospective study that found DSI was not associated with an increased risk of LNM (25). All of these factors led to a significant reduction in the role of DSI in providing treatment decisions for early CRC.

### 2.2 Lymphovascular invasion

Lymphovascular invasion, defined as the invasion of tumor cells into lymphatic vessels or capillaries (30), has also been confirmed to be closely related to LNM in early CRC like DSI (31, 32). Although LVI can be detected by hematoxylin and eosin (H&E) staining, the concordance of assessment is poor due to the high variability between observers. According to Kojima et al. (33), the Kappa values in detecting lymphatic and blood vessel invasion in Japan were only 0.216 (95% CI, 0.133-0.299) and 0.524 (95% CI, 0.441-0.606). Apart from this, another problem is that H&E staining cannot differentiate lymphatic and blood vessels. To solve the problems mentioned above, pathologists attempted to apply other staining techniques, such as D2-40 immunohistochemical staining to detect lymphatic vessel invasion, Victoria Blue (VB), Elastica van Gieson (EVG), CD31, CD34 staining to detect blood vessel invasion (34, 35), but only increased the Kappa value to around 0.50 (36).

### 2.3 Histological grade

Histological grade refers to the differentiation degree of tumor cells, that is, the proximity of tumor cells to normal cells in morphology and function. The higher the differentiation degree of tumor cells, the more similar their morphology and function are to the normal cells from which they originate. Adenocarcinoma is the most common histological type of CRC, which can be divided into three levels: well, moderately, and poorly differentiated. Moderately differentiated can be further subdivided into moderately-well and moderately-poorly differentiated. Compared with well or moderately-well differentiated groups, the risk of LNM in poorly and moderately-poorly differentiated groups was higher (37). This was also confirmed in a recent meta-analysis, which found that patients with poorly differentiated had a 14.61-fold greater risk of LNM than those without this risk factor (38).

### 2.4 Tumor budding

Tumor budding is generally defined as a single tumor cell or cluster consisting of four or fewer tumor cells isolated in the matrix at the front of an invasive tumor. It can be divided into peritumoral budding (PTB, tumor buds at the leading edge of the tumor) and intratumoral budding (ITB, tumor buds at the center of the tumor). Two different methods mainly perform the evaluation. One way is to select the field (0.785 mm²) with the densest budding under 20x objective lens for budding counting and define the field with less than five budding as negative and the field with more than five as positive (39). The other way is to classify the number of lesions by grade: 0-4 lesions are low budding (Bd1), 5-9 lesions are intermediate budding (Bd2), and ≥ 10 lesions are high budding (Bd3) (40). Similar to the pathological factors mentioned above, high budding has been proven to be an independent risk factor for LNM in early CRC (4), but it has not been routinely reported due to the lack of a simple and reproducible standardized scoring system (40).

## 3 New advances in predictive factors of LNM in early colorectal cancer

As mentioned earlier, the treatment of more than 80% of patients is unnecessary if additional surgery is performed solely on the basis of risk factors in the guidelines. Therefore, the new discovery of high-risk factors or evaluation systems is critical.

### 3.1 Newly discovered pathological factors and their advantages

#### 3.1.1 Width of submucosal invasion and area of submucosal invasion

As new predictors, the width of submucosal invasion (WSI) and the area of submucosal invasion (ASI) were also confirmed to be closely related to LNM (10, 41, 42). Combined with Smith’s research mentioned above, ASI was more closely associated with LNM than the DSI (28). However, it has the same trouble as DSI when measuring the submucosal infiltration area, that is, the degree of submucosal invasion is tough to determine. Muscularis mucosae comprises two layers of smooth muscle, inner annular and external longitudinal, located between the mucosal layer and submucosa. To determine the area of invasion of the submucosal layer, the location of the muscularis mucosae must be considered. In practice, however, we often find that the muscularis mucosa is often difficult to identify due to the invasive destruction of the mass. At this point, the WSI appears to be easier to measure, because it only needs to determine the distance between the incision margins of bilateral muscularis mucosa (10).

With the recent application of digital pathology in clinical practice (43–45), the width and area of the tumor invasion can be measured more accurately by implementing automatic algorithms, and it greatly reduces the deviation caused by different observers (46). Once digital pathology is more widely used, the width and area of submucosal infiltration can be more reliable prognostic factors than the DSI in predicting the risk of LNM in early CRC. However, they have not yet determined a uniform cut-off value, which may need to be confirmed by more clinical studies in the future (47).

#### 3.1.2 Poorly differentiated cluster

A poorly differentiated cluster (PDC) comprises ≥5 cancer cells lacking glandular configuration in tumor stroma. Many current studies have confirmed that PDC is a high-risk factor for LNM of early CRC (48, 49). To quantify these PDCs, researchers tend to scan the entire tumor at low magnification to identify the region with the highest number of PDCs at first, then count the clusters at a microscopic field of ×20 objective lens (i.e., a microscopic field with a major axis of 1mm), and finally divide CRC into three levels according to the highest PDC count: <5, 5 to 9 and ≥10 clusters are classified as grade 1 (G1), grade 2 (G2) and grade 3 (G3) (50). Although PDCs are similar to tumor budding in morphology, they are bigger than tumor budding foci by definition. Because of this, we can identify PDC more easily in H&E staining and do not need to rely on immunohistochemical staining techniques (51).

Of course, PDC counting also faces many problems in clinical application, such as the insufficient depth of biopsy sampling position, or mistaking glandular fragments in necrotic or inflammatory areas for PDC. Both mentioned above will lead to inconsistency in PDC evaluation. To solve these problems, we need to exclude the areas showing necrosis or inflammation from counting and take into account the PDC found in all sections (52).

#### 3.1.3 Absence of background adenoma

Background Adenoma (BGA) refers to benign adenomatous tissue adjacent to cancerous tissue. CRC mainly occurs in two ways: one is developed from an adenomatous polyp through the adenoma-carcinoma sequence, and the other is from scratch without the precursor stage of an adenomatous polyp (53). BGA is considered to be the primary focus to complete adenoma-adenocarcinoma transformation in early CRC, therefore, we regard the absence of BGA as the histological feature of the primary cancer (54). Through retrospective analysis, Suh et al. found that the absence of BGA is a risk factor for predicting LNM in T1 CRC (55), which was also confirmed in Han et al. ‘s study (56). The molecular genetic mechanism related to primary cancer is still unclear at present yet, the absence of BGA cannot be excluded from the rapid growth of tumors, thus more research is needed in the future.

### 3.2 Clinical test parameters related to LNM

#### 3.2.1 Fibrinogen

**Figure 2:**
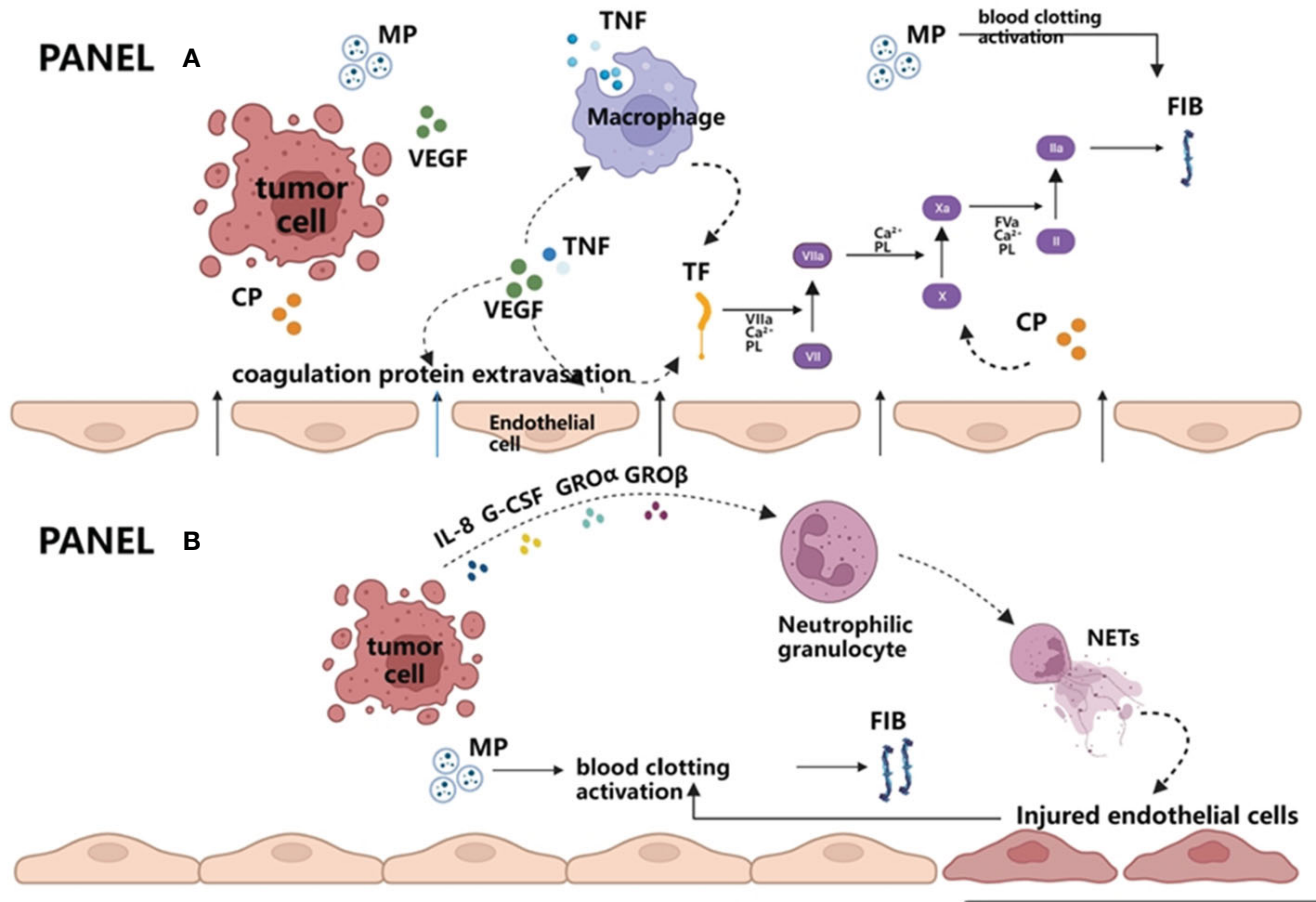
Extravascular and intravascular activation of blood coagulation by tumor cells. **(A)** Extravascular activation of blood coagulation by tumor cells. Vascular endothelial growth factor (VEGF) produced by tumors and/or tumor necrosis factor (TNF) produced by macrophages destroy the vascular permeability barriers on the one hand and lead to FIB and other required coagulation protein substrates entering the extravascular space. On the other hand, both cytokines can activate procoagulant tissue factor (TF) in endothelial cells and macrophages, thus initiating exogenous coagulation reactions and producing FIB. In addition, MP released by tumor cells can also enhance the coagulation cascade, which eventually leads to thrombin generation and fibrin formation. **(B)** Intravascular activation of blood coagulation by tumor cells. Once tumor cells enter the blood circulation, granulocyte colony stimulating factor (G-CSF) and interleukin-8 (IL-8) produced by tumor cells can cooperate with GRO α or GRO β, promoting neutrophils to release NETs. NETs are DNA network structures and are decorated with histones, granular proteins, etc. They may serve as the initial link between the immune system and coagulation system, directly participate in promoting the formation of microthrombus, or further activate coagulation reaction by damaging endothelial cells. Apart from this, it is similar to the extravascular coagulation pathway in that tumor cells in circulating blood can also activate blood clotting by releasing MP. VEGF, vascular endothelial growth factor; TNF, tumor necrosis factor; TF, tissue factor; MP, microparticles; FIB, fibrinogen; NETs, neutrophil extracellular traps.

At present, many studies have found that hemostatic factors are closely related to cancer growth and metastasis. Many molecular mechanisms cause the hypercoagulable state of cancer patients themselves. Whether it is the activation of extravascular coagulation by tumor cells or the activation of intravascular coagulation, the interaction of tumor cells with vascular endothelium and components of the coagulation cascade is inseparable (**Figure 2**) (57, 58).

Fibrinogen (FIB), one of the most abundant plasma coagulation proteins, was first observed by Bilrotte to be enriched around tumor cells (59). Extensive experimental evidence also points out the role of FIB in advanced CRC. Joseph found a significantly lower incidence of pulmonary and regional lymph node metastases in spontaneous hematogenous and lymphatic metastasis in mice with FIB deficiency (60). This conclusion has also been verified in humans. The retrospective studies by Wang and Berrin et al. all found higher FIB levels in patients with LNM in CRC than in patients without LNM (61, 62).

Of greater interest is the fact that pharmacological inhibitors associated with the coagulation system have been shown to significantly reduce the probability of tumor metastasis in animal studies (63). This means that the measurement of FIB level to predict LNM in early CRC can not only make a decision on the surgical options, but also provide a reference for the selection of candidates suitable for early drug intervention in the coming future.

#### 3.2.2 Fatty acid-binding protein 4

As the basic components of biofilms, lipids not only play an important role in energy storage and metabolism, but also promote the rapid proliferation and metastasis of cancer cells in the tumor microenvironment (64). Even in the tumor microenvironment lacking oxygen and nutrients, cancer cells can accumulate large amounts of lipids through reprogramming of lipid anabolic metabolism (e.g., up-regulation of key regulators of lipogenesis, such as sterol regulatory element-binding proteins (SREBPs), acetyl-CoA carboxylase (ACC), fatty acid synthase (FASN) and stearoyl-CoA A desaturase 1 (SCD1)) (65, 66), thus providing cell membranes and organelles for cancer cells to proliferate (67). In addition, tumor cells can also activate lipolysis in adipocytes to decompose triglycerides into fatty acids, which are transported to tumor cells via fatty acid transporters for β-oxidation energy supply, ultimately promoting tumor progression (68).

Fatty acid-binding protein 4 (FABP4) is a carrier protein of fatty acids, which is widely expressed in adipocytes, monocytes, and macrophages, and is responsible for participating in lipid transport, metabolism, and intracellular signal transduction (69). FABP4 levels have been associated with breast cancer (70), ovarian cancer (71), prostate cancer (72) and cholangiocarcinoma (73). In recent years, an increasing number of experiments have found significantly higher plasma levels of FABP4 in patients with CRC as well (71, 74). At the same time, a link between FABP4 and tumor metastasis has been continuously reported. Zhang et al. showed that plasma FABP4 level was negatively correlated with LNM in CRC (75), which seems to be contrary to our previous views. The reason for this result may be that compared with the patients with CRC without LNM, patients with LNM tend to require more plasma FABP4 to participate in lipid transport and metabolism. Of course, not all studies support this view (74), and the association between plasma FABP4 level and LNM of early CRC is not yet clear. For this reason, more clinical data are needed to verify and support it in the future.

### 3.3 Molecular biomarkers associated with LNM

In addition to pathological and clinical test factors, molecular biomarkers can also be predictors of LNM in early CRC. According to the biological origin of the material, we can classify molecular biomarkers into tumor tissue-based or blood-based molecular biomarkers.

#### 3.3.1 Tumor tissue-based molecular biomarkers

MicroRNAs (miRNAs) are a class of small non-coding RNA molecules that regulate gene expression (76). Over the past decade, numerous studies have revealed the functions of miRNAs in CRC progression (77). Ozawa et al. (78) found that five miRNAs (MIR32, MIR181b-1, MIR193b, MIR195, and MIR411) were differentially expressed between tumor tissues in the lymph node metastasis group and non-metastasis group by using the database from the Cancer Genome Atlas (TCGA). When combined with the above miRNAs to predict LNM, the discovery, training, and validation cohort all had good discrimination, and their area under curve (AUC) was 0.840, 0.830, and 0.740, respectively. Kandimalla et al. (79) then screened out eight mRNAs (AMT, MMP9, FOXA1, LYZ, MMP1, C2CD4A, PIGR, RCC1) from the genome-wide mRNA-expression database to form a new prediction model, which was confirmed to have good prediction ability as well.

Caudal type homeobox transcription factor 2 (CDX2) is a protein expressed in the nucleus of intestinal epithelial cells, which plays an important role in the embryonic formation and differentiation of the intestine ( (80)). It is precisely because the transcription of CDX2 is limited to colon and small intestine cells that CDX2 is often used as a specific immune marker for CRC (81). CDX2 is considered to be a tumor suppressor gene in CRC and patients lacking CDX2 are more likely to develop LNM ( (82)), but whether it has the same value in early CRC needs further research and exploration.

#### 3.3.2 Blood-based molecular biomarkers

Compared with tumor tissue-based molecular biomarkers, the convenience of blood collection or donation means that the detection of blood biomarkers may become a more practical screening tool for predicting LNM in early CRC.

Circulating tumor cells (CTC) are epithelial cancer cells from primary or metastatic tumors that enter the circulatory system and can be detected in peripheral blood (83). Pan et al. found that the probability of CTC positive in CRC patients with LNM was significantly higher than that of patients without LNM (84), but due to the low blood concentration of CTC, especially when the tumor is in the early stage, this method has limitations as a diagnostic tool for LNM in early CRC. It is expected to make up for this defect by improving the detection methods of CTC in the future (83).

The relationship between miRNA, mRNA, and LNM in early CRC has been confirmed in tumor tissue. To use these indicators for preoperative evaluation, Wada et al. (85) converted these molecular biomarkers into blood-based noninvasive detection and finally obtained a new model consisting of four miRNAs (miR-181 b, miR-193 b-3 p, miR-195 p, and miR-411-5 p) and five mRNAs (AMT, FOXA 1, MMP 1, MMP 9 and PIGR). The AUC of the combination of molecular markers in the training and validation cohort were 0.860 and 0.820. The research also has limitations: it is a retrospective analysis and includes a few positive cases. In the future, a prospective clinical trial with a larger sample size is needed to further verify the effectiveness of the model.

Long non-coding RNAs (lncRNAs) are non-protein-coding RNAs with a length of more than 200 nucleotides (86), and because lncRNAs can cross cell membranes, they can be found in the blood (87). They act as powerful regulators of gene function and cellular processes and are involved in the proliferation, growth, apoptosis, invasion, and metastasis of tumor cells (88). Many lncRNAs have been found to be closely related to LNM in CRC, including colon cancer-associated transcript1 (CCAT1), differentiation-antagonistic non-protein coding RNA (DANCR), HOX transcript antisense intergenic RNA (HOTAIR) and metastasis-associated lung adenocarcinoma transcript1 (MALAT1) (89–93). Among them, the nuclear paraspeckle assembly transcript 1 (NEAT1), which is the target gene of P53, is a key part of the composition of the para-spot structure (94). Prospective studies by Li et al. found that CRC patients with high expression of NEAT1 had a significantly higher probability of LNM than patients with low expression of NEAT1 (95). As a type of lncRNA, plasmacytoma variant translocation 1 (PVT1) was also found to be significantly associated with LNM in CRC (96). And when it was combined with HOTTIP and UCA1, they exhibited higher accuracy (97). To sum up, lncRNA has the potential to become a biomarker for predicting LNM in early CRC.

### 3.4 Predictive scoring system

The discriminating power of these models was poor when the risk factors provided in the guidelines were used to predict LNM of early CRC (ASGE/ESGE, AUC=0.670; JSCCR, AUC=0.640) (13). However, when we incorporated newly discovered pathological or other relevant factors into the new models, we found that their discrimination ability was significantly improved (**Table 2**).

**Table 2:** Predictive scoring systems for LNM of patients with T1 colorectal cancers.

Author [year]	Country	No. of patients	Risk factors	OR [95% CI]	AUC
Miyachi et al. [2016] (98)	Japan	653	Sex Lymphovascular infiltration Tumor budding Por/Muc component	2.220 [1.260–3.910] 9.840 [3.420–28.300] 1.800 [1.010–3.210] 2.310 [1.250–4.270]	/
Oh et al. [2019] (8)	Korea	833	Histologic grade Submucosal invasion Background adenoma Vascular invasion Tumor budding	7.890 [2.890-21.520] 2.140 [1.190-3.860] 0.580 [0.360-0.920] 8.450 [4.560-15.660] 1.700 [1.030-2.800]	0.812
Yan et al. [2019] (99)	China (data from SEER)	21880	Age [ref; <45 y] 45-65 y ≥65 y Marriage [ref; married] Single Unknown CEA [ref; negative] Borderline Positive Unknown Histological type [ref; adenocarcinoma] Carcinoid tumor Neuroendocrine carcinoma Mucinous adenocarcinoma Other T classification Histological grade [ref; well differentiated] Moderately differentiated Poorly differentiated Undifferentiated Tumor size [ref; <5 cm] ≥ 5 cm Unknown	0.830 [0.692-0.996] 0.525 [0.438-0.630] 0.898 [0.826-0.976] 0.806 [0.675-0.962] 1.468 [0.743-2.900] 1.385 [1.228-1.561] 0.740 [0.678-0.808] 1.752 [1.328-2.311] 3.740 [2.613-5.534] 1.046 [0.881-1.241] 1.118 [0.933-1.339] 2.221 [2.030-2.431] 1.644 [1.442-1.875] 3.641 [3.088-4.292] 3.462 [2.609-4.593] 1.125 [1.003-1.262] 0.840 [0.731-0.967]	0.667
Guo et al. [2020] (100)	China (data from SEER)	17309	Age at diagnosis [ref; 18-49] 50-64 65-79 80+ Race [ref; white] Black Asian/Pacific Islander American Indian/Alaska Gender Marital status [ref; married] Unmarried Unknown Tumor location [ref; right side] Left side Not stated Histology [ref; adenocarcinoma] Mucinous adenocarcinoma Other/Not stated Tumor size [ref; 1-9 mm] 10-19 mm 20-29 mm 30 + mm Not stated Grade [ref; well differentiated] Moderately differentiated Poorly differentiated Undifferentiated Not stated CEA [ref; positive] Negative Borderline/Unknown	0.860 [0.750-0.990] 0.610 [0.530-0.710] 0.460 [0.370-0.570] 1.110 [0.960-1.290] 1.190 [1.020-1.390] 0.790 [0.380-1.460] 0.810 [0.740-0.890] 0.900 [0.820-1.000] 0.780 [0.620-0.970] 1.590 [1.430-1.760] 0.860 [0.690-1.070] 2.190 [1.700-2.800] 1.920 [1.250-2.890] 1.240 [1.070-1.440] 1.170 [0.990-1.370] 1.560 [1.340-1.810] 0.950 [0.920-1.110] 1.760 [1.530-2.040] 3.990 [3.310-4.810] 2.330 [1.500-3.530] 1.140 [0.910-1.430] 0.830 [0.700-0.990] 0.650 [0.550-0.780]	0.666
Mo et al. [2020] (11)	China (data from SEER)	8363	Age Tumor site Tumor grade [ref; I] II III-IV T stage pre-CEA [ref; negative] positive other cLNM Perineural invasion	0.655 [0.570-0.753] 1.325 [1.152-1.525] 1.744 [1.382-2.200] 4.445 [3.367-5.870] 1.899 [1.650-2.185] 1.283 [1.041-1.580] 0.777 [0.675-0.896] 18.081 [12.736-25.670] 3.566 [2.571-4.947]	0.720
Gambella et al. [2022] (101)	Italy	207	Age Lymphovascular invasion TILs Tumor budding	0.260 [0.090–0.710] 23.80 [5.12–110.90] 0.190 [0.060–0.590] 5.210 [1.600–16.800]	/
Kajiwara et al. [2023] (102)	Japanese	4673	Sex Location [ref; T] A/C/D S/Rb Rs/Ra Tumor grade [ref; G1] G2 G3 Lymphovascular invasion Tumor budding SM invasion depth [ref; <1000] 1000-1999 ≥2000	1.550 [1.170–2.050] 1.740 [0.880–3.450] 2.180 [1.140–4.150] 2.590 [1.310–5.120] 1.730[1.290-2.310] 3.600[1.660-7.820] 3.050[2.200-4.230] 1.930[1.430-2.610] 3.000[1.510-5.950] 4.330[2.370-7.940]	0.784

OR, odds ratio; CI, confidence interval; SEER, Surveillance, Epidemiology, and End Results; Por/Muc; poorly differentiated adenocarcinoma or mucinous carcinoma; CEA, carcinoembryonic antigen; cLNM, clinical lymph node metastasis; TILs, tumor-infiltrating lymphocytes; A, ascending colon; C, cecum; D, descending colon; S, sigmoid colon; Rb, lower rectum; RS, rectosigmoid; Ra, upper rectum.

In tumor grade: I means well-differentiated; II means moderately differentiated; III means poorly differentiated and IV means undifferentiated; G1 means papillary adenocarcinoma and well-differentiated tubular adenocarcinoma, G2 means moderately differentiated adenocarcinoma; G3 means poorly differentiated adenocarcinoma, mucinous adenocarcinoma, or signet ring cell carcinoma. The / indicates this model are not mentioned in the article.

Oh et al. (8) collected patients with T1 CRC who underwent endoscopic or surgical resection at the National Cancer Center, and incorporated five significant independent risk factors in multivariate analysis (vascular invasion, histological grade, submucosal invasion, tumor budding, and BGA) into the prediction model. It is concluded that the AUC of the model was 0.812 in the development cohort and 0.771 in the validation cohort. Kajiwara et al. (102) also created a prediction model with AUC up to 0.784 which included six clinical and pathological factors, such as gender, tumor location, histological grade, LVI, tumor budding, and submucosal invasion. Nevertheless, these studies also have limitations. On the one hand, the risk factors mentioned in these models are inconsistent, and some newly discovered meaningful pathological or clinical test factors are not included. On the other hand, these studies apply retrospective analysis and may lead to bias. Therefore, multi-center prospective studies with large samples are needed to further develop and verify these models in the future.

### 3.5 Artificial intelligence system

Artificial intelligence (AI) is the result of the evolution of general software systems which allows for decision-making that mimics human intelligence (103). As a sub field of AI, machine learning (ML) enables a machine to become more effective with training experience (104). In recent years, there has been a growing interest in the development of MI applications for predicting LNM of early CRC. As early as 2018, Kudo confirmed the feasibility of artificial intelligence to predict LNM. He found that compared with current guidelines, the artificial intelligence model significantly reduced unnecessary surgery after endoscopic resection of T1 CRC (105). However, the credibility of AI’s diagnostic performance declined because the data it learned and verified came from the same institution, and it did not include cases undergoing endoscopic resection alone.

In order to overcome these limitations, Kudo et al. (106) collected clinicopathological information (age, sex, tumor size, location, morphology, LVI, histological grade, and corresponding LNM) of T1 CRC patients from six hospitals in Japan, constructed a prediction model through machine-learning artificial neural network (ANN) and verified it in T1 CRC patients in another hospital in the same period. It was found that the AUC of the ANN model was 0.830, and its diagnostic ability was also better than the guidelines’.

In addition to incorporating clinical features into the AI model, Song et al. scanned the endoscopic resection specimens of patients undergoing extra surgery after endoscopic treatment with H&E-stained whole slide images (WSIs) and developed a new AI prediction model by using a two-step attention-based deep learning approach (107). The final model showed higher prediction accuracy than the traditional pathological model. Moreover, the AI system can automatically extract specific features from images without human intervention (108). It not only accelerates pathological analysis, but also greatly reduces the misdiagnosis caused by fatigue or distraction of pathologists (109).

At present, the use of AI systems to predict LNM is still in the stage of research and exploration, but we believe that in the near future, AI technologies will play a more important role in the actual clinical application of CRC.

## 4 Summary

With the development and maturity of endoscopic technology, the number of patients with early CRC cured by endoscopic surgery is increasing. Therefore, there is no clear conclusion in the current guidelines whether patients with T1 CRC who undergo endoscopic resection need additional surgery plus regional lymph node dissection. But what we can be sure of is that if LNM is present in T1 CRC, then the clinical benefit of second surgery is great (11). As the number of patients with T1 CRC will increase in the future, accurate assessment of LNM status will be critical in making decisions about treatment options for this population. At present, it is rare to use a certain factor to predict the risk of LNM in pT1 CRC alone. With the development of various scoring systems, the accuracy and sensitivity of prediction have been substantially improved.

However, most of the parameters included in the current predictive scoring systems are pathological parameters, such as histological type, lymphatic vascular invasion, depth of submucosa (SM) invasion, and tumor budding (**Table 2**) (8, 11, 98–101). The AUC of these models tends to be between 0.650 and 0.700, indicating that the discrimination ability of these models is average. In addition, recent studies have found that DSI has nothing to do with an increased risk of LNM, which indicates that we need to incorporate some new meaningful parameters to improve the accuracy of the prediction model.

This review focuses on the new predictors associated with LNM in early CRC reported in recent studies. Although WSI and ASI are also pathological parameters, they have shown more reliable predictive power than DSI as new predictors (28). While the measurement of ASI has the same troubles as DSI, the development and application of digital pathology will solve this problem well for the time to come (46). The detection of PDC is more convenient than tumor budding. As a risk factor for LNM in T1 CRC, the absence of BGA has also been demonstrated in recent studies. In addition to pathological factors, clinical test factors are also associated with LNM. FIB, as the final product of the coagulation cascade, has also been proven to be highly expressed in CRC with LNM. As one of the routine preoperative tests, FIB data are easy to collect, but it is subject to many confounding factors, such as anticoagulant use, auto-thrombosis, and viral infection, which can also be greatly attenuated under strict inclusion criteria. Evidence for the relationship between FABP4 and LNM in CRC is limited, but it is closely related to tumor progression (75), and it is believed that the relationship between FABP4 and LNM will become clearer in the future with the corroboration of more clinical data. Apart from pathological and clinical test factors, molecular biomarkers can also be predictors of LNM in early CRC. According to the biological origin of the material, we can classify molecular biomarkers into tumor tissue-based or blood-based molecular biomarkers. Many studies have confirmed their association with LNM in CRC, but whether they have the same value in early CRC needs further research to explore. At the same time, due to the explosive growth of clinical data, AI has shown great advantages and application potential in the prediction of LNM of early CRC. A large number of studies have found that the prediction model constructed by ML shows a more excellent and stable prediction effect (105–107).

In conclusion, the number of new predictors and models that have been reported is on the increase, but whether they can accurately predict the risk of LNM in T1 CRC requires more retrospective or prospective studies to verify.

## Author contributions

XS: Conceptualization, Writing – original draft, Writing – review & editing. JL: Conceptualization, Writing – review & editing. JiaZ: Conceptualization, Writing – review & editing. Y-FK: Conceptualization, Writing – review & editing. Y-HZ: Conceptualization, Writing – review & editing. Z-KW: Conceptualization, Writing – review & editing. JinZ: Conceptualization, Writing – review & editing.
